# A Bibliometric Analysis of Research on Appearance Anxiety

**DOI:** 10.7759/cureus.90364

**Published:** 2025-08-18

**Authors:** Li Yang, Shixin He, Xiao Qin

**Affiliations:** 1 Department of Medical Cosmetology, The Third People's Hospital of Chengdu, Chengdu, CHN

**Keywords:** appearance anxiety, bibliometrics, bibliometrix, frontiers, hotspots

## Abstract

Over the past two decades, research on appearance anxiety has experienced a significant surge. There is an urgent need to sort out these studies to promote the integration of the discipline. This study conducted a bibliometric analysis of research related to "appearance anxiety" in the Web of Science Core Collection (WoSCC) via a visualization tool, aiming to clarify the research hotspots and frontiers, and provide valuable information for researchers in this field. A total of 365 publications were searched. The expansion of research scale in this field coexists with the dilution of academic influence. The USA is the dominant country in this field. International collaboration urgently needs to be strengthened. The research hotspots focus on studies related to appearance anxiety and related psychological and social aspects. They are concentrated on issues related to body image and mental health among the adolescent population. The research frontiers not only delve deeper into the individual but also examine the long-term impact of appearance anxiety on an individual's social functions, interpersonal relationships, and overall quality of life.

## Introduction and background

Appearance anxiety refers to a psychological state where an individual experiences persistent distress due to excessive concern about their physical flaws, resulting in impairment of social, professional, or other functions [1,2]. This psychological phenomenon not only triggers intense self-denial and emotional distress but also may lead to social withdrawal, decreased professional performance, and even breakdowns in interpersonal relationships. It has been reported that individuals with appearance anxiety often get trapped in a vicious cycle of excessive grooming or social avoidance, and some may even develop depression [3].

Furthermore, appearance anxiety is associated with body dysmorphia and eating disorders [4-6]. Still, it also involves the broader pressure to conform to the appearance standards imposed by social media and the aesthetic culture [7]. Currently, scales are commonly used as tools for evaluating body image anxiety. For instance, the Social Appearance Anxiety Scale is a specific tool designed to quantify the anxiety emotions that individuals experience due to their appearance in social situations, and it is mainly used to explore the influencing factors of social appearance anxiety [8,9]. Furthermore, the Appearance Anxiety Inventory measures the overall concerns of individuals regarding their appearance more broadly and is not specific to social situations [10,11]. Over the past two decades, research on appearance anxiety has experienced a significant surge. However, the research directions have been scattered, with significant differences in regions, methods, and theoretical frameworks. There is an urgent need to sort out these issues to promote the integration of the discipline.

The bibliometric method quantitatively analyzes publication trends, interdisciplinary connections, and knowledge gaps, providing unique value for a comprehensive depiction of the research landscape [12]. Compared to traditional reviews, its advantage lies in the application of techniques such as citation analysis and co-occurrence networks, which enable the identification of high-impact scholars and emerging fields at a macro level, as well as the visualization of collaboration networks and their temporal evolution. Therefore, this study conducted a bibliometric analysis of research related to "appearance anxiety" in the Web of Science Core Collection (WoSCC) via the visualization tool Bibliometrix [13], aiming to clarify the research hotspots and frontiers and provide valuable information for researchers in this field.

## Review

Methods

This study utilized the WoSCC as the primary data source, which includes the Science Citation Index Expanded (SCIE), Social Sciences Citation Index (SSCI), and Arts and Humanities Citation Index (AHCI), to ensure comprehensive coverage of interdisciplinary literature. The detailed search strategy was as follows: "TS= "appearance anxiety" OR "appearance anxiousness" OR “appearance anxiet*" OR "appearance anxi*" (period: 2006-01-01 to 2025-06-30). The last retrieval date was July 16, 2025. To ensure the comprehensiveness of the included literature, no restrictions are imposed on the language or type of articles in the search. We used Bibliometrix version 5.0 to remove duplicate entries from the exported data and conduct bibliometric analysis, which summarized the annual publication trends, author and journal networks, and keyword co-occurrences. To access this, we used RStudio 2025.05.1 Build 513 (Posit Software, PBC, Boston, MA). All parameters are selected from the built-in parameters of Bibliometrix.

Results

Publication Trends

**Figure 1:**
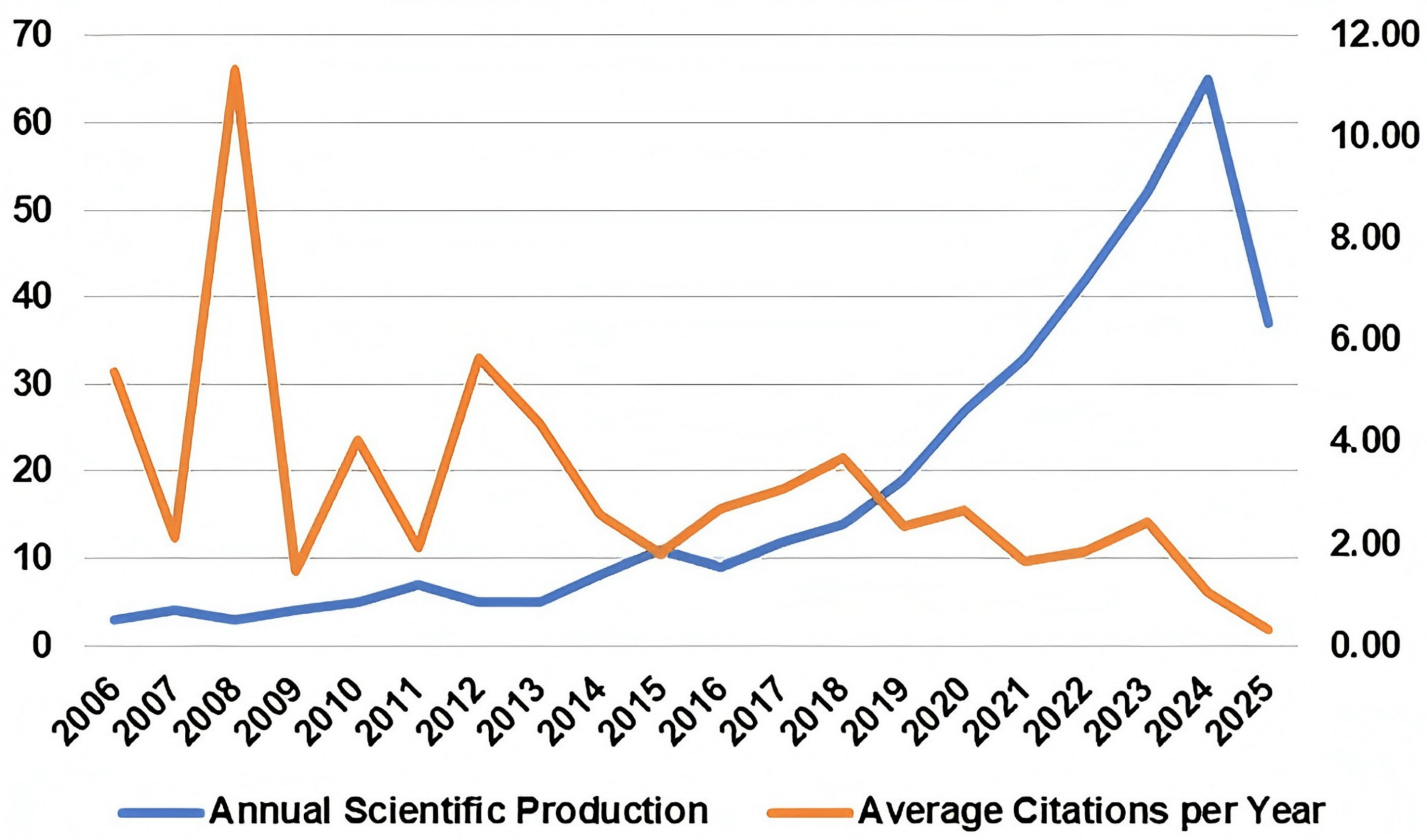
Publications and citations

A total of 365 publications were searched. Between 2006 and 2024, the number of publications exhibited a continuous and significant growth trend. Since 2010, the annual growth rate has increased significantly, and by 2024, the number of publications had risen to 65, more than 20 times the number in 2006. The average citation frequency showed a significant downward trend. It reached a peak of 11.33 times in 2008 and then dropped sharply to 1.46 times in 2009. Since then, except for a brief recovery in 2012 (5.64 times) and 2018 (3.70 times), the overall trend has continued to decline (Figure 1).

Journals

A total of 230 journals have published research in this field. The top three journals, ranked by publication volume, are Body Image, Current Psychology, and Sex Roles (Table 1). Additionally, Body Image was the journal with the highest H-index, G-index, and M-index. Although Current Psychology had a higher number of publications, its citation indicators have decreased relatively.

**Table 1:** Top 10 journals in this field TC: total citations, NP: number of publications

Journal	H-index	G-index	M-index	TC	NP
Body Image	12	20	0.632	528	20
Current Psychology	4	8	0.571	68	12
Sex Roles	7	8	0.35	692	8
Child Psychiatry & Human Development	4	7	0.4	60	7
Frontiers in Psychology	3	4	0.3	16	6
Psychology of Women Quarterly	5	5	0.357	312	5
Assessment	4	5	0.222	299	5
BMC Psychology	2	3	1	12	5
Appetite	4	4	0.308	209	4
Eating and Weight Disorders - Studies on Anorexia, Bulimia and Obesity	4	4	0.267	85	4

Countries

**Figure 2:**
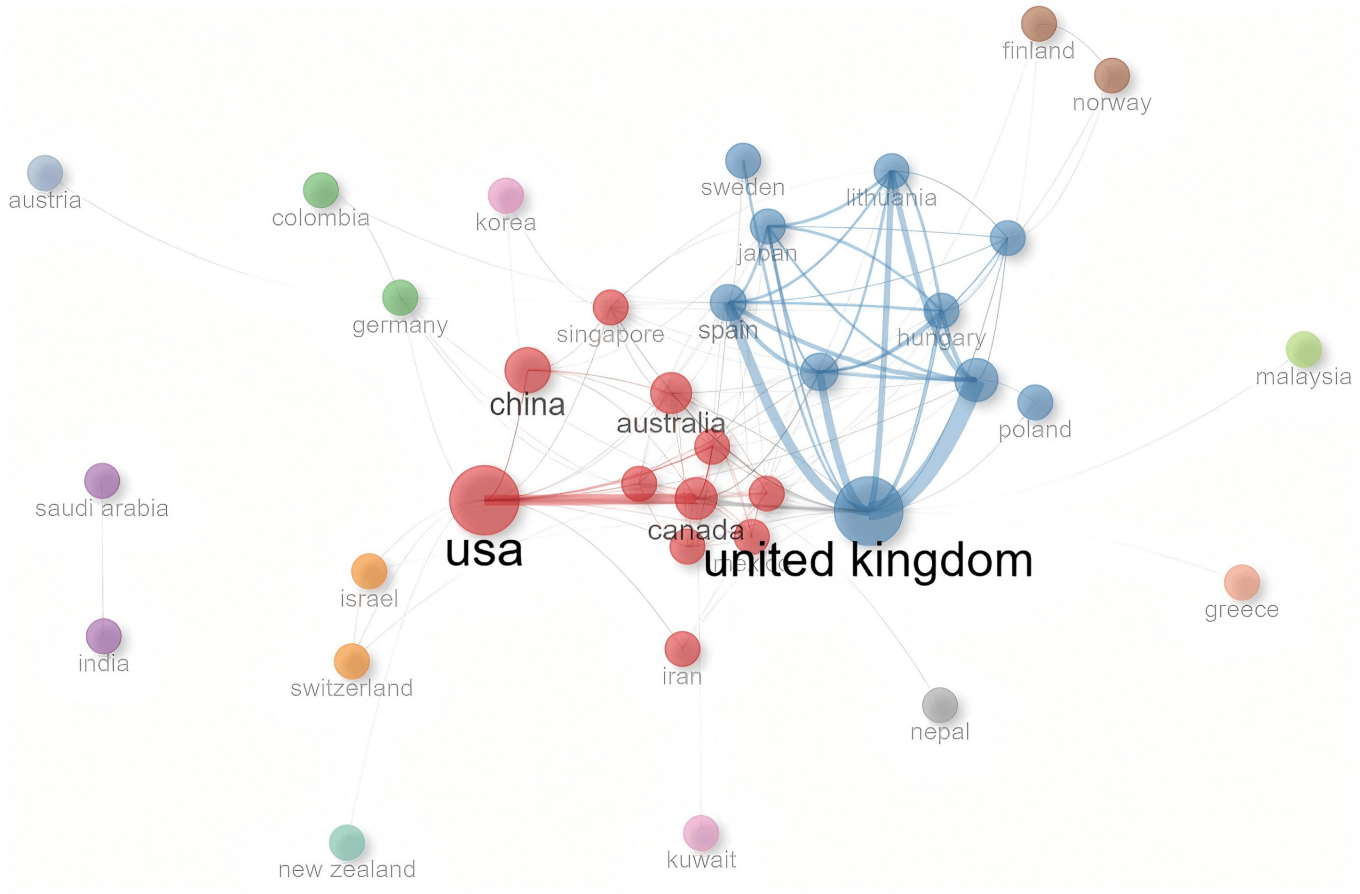
Country collaboration network

A total of 41 different countries published articles in this field (Table 2). The USA had the highest number of articles in this field, with 60, significantly surpassing other countries. Turkey and China followed closely, publishing 52 and 46 articles, respectively, demonstrating strong research capabilities. In the country collaboration network, the Betweenness of the United Kingdom was as high as 171.866, far exceeding other countries, playing a key role as a critical bridge in international scientific research collaboration; the PageRank value of the USA was 0.069, with overall influence leading the pack; Canada's Closeness was 0.022, with a higher degree of connection with other countries (Figure 2).

**Table 2:** Top 10 countries, affiliations, and authors *The statistics are based on the countries/regions where the corresponding authors of the articles are from.

Country*	Articles	Affiliation	Articles	Authors	Articles
USA	60	University of London	28	Levinson CA	15
Turkey	52	Griffith University	25	Zimmer-Gembeck MJ	12
China	46	Griffith University - Gold Coast Campus	22	Corazza O	10
Australia	35	King's College London	20	De Luca I	8
United Kingdom	26	McGill University	20	Krebs G	8
Canada	19	University of Louisville	17	Martinotti G	8
Italy	17	University of California System	16	Simonato P	8
India	7	South London and Maudsley NHS Trust	15	Burkauskas J	7
Iran	6	Universidade do Porto	13	Farrell LJ	7
Cyprus	4	University of Hertfordshire	13	Jassi A	7

Affiliations

**Figure 3:**
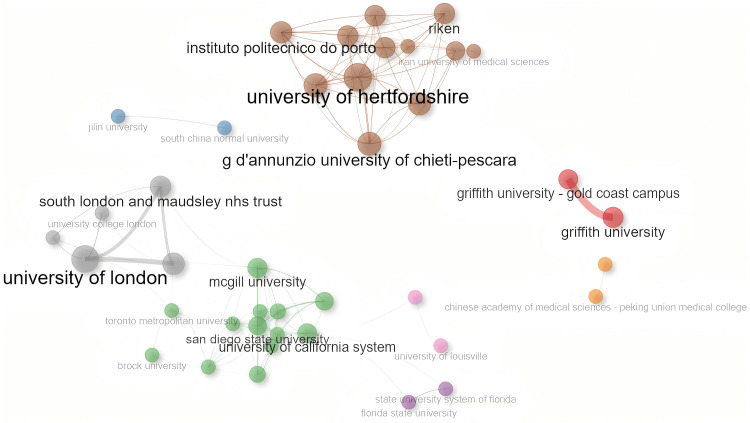
Affiliation collaboration network

A total of 528 affiliations published articles in this field (Table 2). The University of London and Griffith University published the most articles, with 28 and 25, respectively, and they were important research forces in this field. Other affiliations, such as Griffith University - Gold Coast Campus and King's College London, also have a certain number of article outputs. From the perspective of the affiliation collaboration network, multiple affiliations form different clusters. In the cluster with the largest number of institutions, the Betweenness of the University of California System was the highest at 72; McGill University's PageRank value was 0.039, with overall prominent influence, while most institutions had these two values of 0, and their roles in collaboration were relatively limited (Figure 3).

Authors

**Figure 4:**
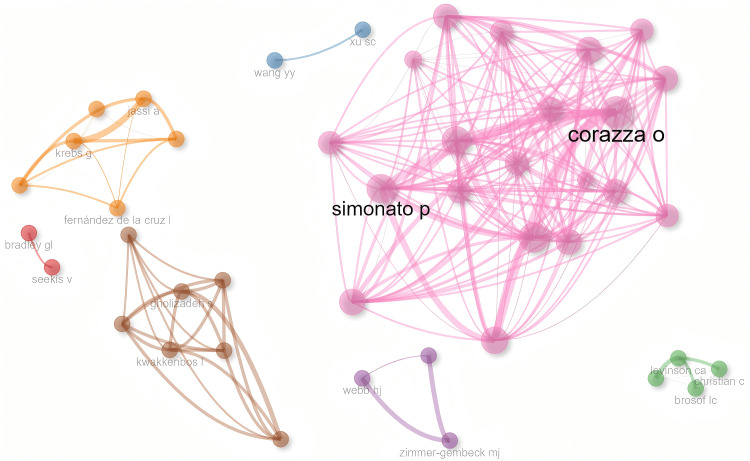
Author collaboration network

A total of 1,305 authors published articles in this field (Table 2). Levinson CA published the most articles, with 15, making the author a representative scholar. Zimmer-Gembeck MJ, Corazza O, and other authors also published a considerable number of articles, demonstrating their activity and contribution. From the overall author collaboration network perspective, the Betweenness of most authors was 0. Seekis V, Bradley GL, Wang YY, and Xu SC have a Closeness of 1, indicating that they were relatively closely connected with others (Figure 4).

Keywords Analysis

**Figure 5:**
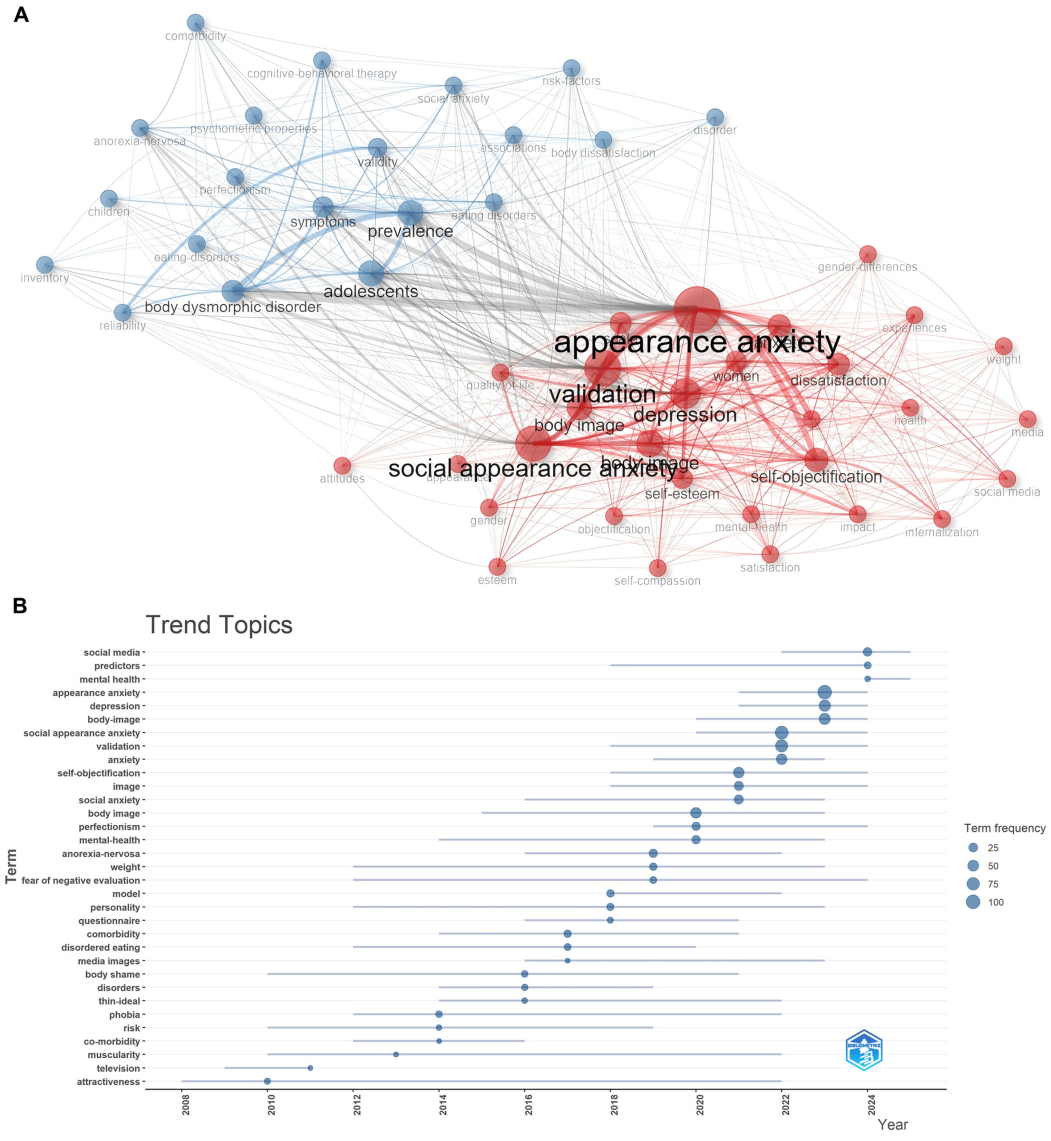
Keywords analysis (A) Keywords co-occurrence. (B) Trend Topics analysis.

We further analyzed the keywords in this field to clarify the research hotspots and frontiers. A total of five key terms appeared more than 50 times (Table 3). Cluster analysis further divided the key terms into two clusters (Figure 5A). Cluster 1 mainly focused on "appearance anxiety", covering multiple related key terms such as "social appearance anxiety", "body image", and "self-objectification". Cluster 2 centered on "adolescents" and is associated with key terms such as "prevalence", "body dysmorphic disorder", and "symptoms". Trend Topics analysis further revealed that the popularity of high-frequency words such as "body image" and "self-objectification" has continued to rise in recent years, and the research span was long. Emerging hotspots such as "social appearance anxiety" and "appearance anxiety" had high frequencies, and their earliest research years were later. Although "mental health" had a low frequency, its earliest research year was the latest (Figure 5B).

**Table 3:** Top 10 keywords

Words	Occurrences
Appearance anxiety	104
Social appearance anxiety	87
Validation	74
Depression	61
Body image	55
Self-objectification	47
Anxiety	46
Body image	46
Adolescents	43
Prevalence	43

Discussion

In the field of appearance anxiety, the number of articles published each year has been growing steadily, indicating a rapid increase in research activity in this area. However, in the later stage of the research (2020-2024), the average citation rate was less than twice per year, suggesting that this field may face research saturation or a shift in academic attention. The contradiction between the sharp increase in publication volume and the decline in citation rate suggests that there is both an expansion of research scale and a dilution of academic influence in this field, as well as an influx of low-quality research and fragmentation of themes, which dilutes academic influence.

Journal analysis can provide data support for scholars' submission strategies, institutional resource allocation, and policymakers' tracking of the academic ecosystem. We found that body image has a significant academic influence and high-quality research output in this field. Although BMC Psychology publishes a relatively small number of articles, the average influence of each article is relatively strong. Research on appearance anxiety reveals significant geographical distribution differences. The USA has the highest number of published papers, followed by Turkey and China. English-speaking countries (the USA, the United Kingdom, Australia, and Canada) collectively contribute the majority of papers. The research capabilities in non-English-speaking countries are relatively weak. Moreover, the individual contributions within the field are highly concentrated. Levinson CA and Zimmer-Gembeck MJ are the leading authors. At the institutional level, the University of London and Griffith University are the global core research centers, highlighting the clustering advantages of British universities.

The USA and the United Kingdom lead global collaboration. Canada, although with a relatively low number of publications, serves as a bridge between the research clusters of the USA and the United Kingdom. China and Australia have close internal collaboration but limited cross-regional cooperation. European countries, such as Italy and Spain, and Latin American countries, such as Colombia and Brazil, form a regional closed network. India, Saudi Arabia, France, and the Netherlands lack cross-border interaction. The situation of institutional collaboration is similar. The core hubs in North American institutions, such as McGill University and the University of California system, dominate cross-regional collaboration through their intermediary centrality. European institutions have close internal collaboration but lack transcontinental bridge collaboration. The intermediary centrality of Asian institutions is close to zero, turning into isolated nodes. Most scholars have close internal partnerships but lack direct connections with scholars from other regions. Moreover, although the high-yield scholar group produces intensively, their collaboration is also limited to closed sub-networks, lacking interdisciplinary or cross-regional knowledge flow.

Keyword analysis can further reveal the research hotspots in this field. Cluster analysis indicates that currently, there are two main research hotspots in this domain: (1) studies on appearance anxiety and its related psychological and social aspects, covering different manifestations of appearance anxiety, influencing factors, and their correlations with other psychological concepts, such as self-objectification and body image [14,15], and issues related to body image and mental health among the adolescent population, including symptom manifestations and psychological measurement characteristics, aiming to gain a deeper understanding of the physical and mental health challenges faced by adolescents at a specific stage [2,16].

In addition, the results of Trend Topics also further reveal that researchers have focused their research direction on the anxiety emotions that individuals experience due to their appearance in social situations. This anxiety is no longer confined to the individual; it has become a collective concern. Still, it extends to various scenarios such as social interactions and public displays, delving into its formation mechanism, influencing factors, and long-term effects on the individual's social functions, interpersonal relationships, and overall quality of life [17,18]. For example, the prominent manifestation of the term "self-objectification" reflects a deep exploration of the individual's internal psychological cognitive level from a research perspective [15,19,20].

This study has some limitations. The database is incomplete and has a language and regional bias, which may lead to the omission of some research results and result in biased analysis [21]. Results can be skewed toward well-indexed, Western, English journals. Moreover, the selection of bibliometric indicators and the parameters of visualization tools can introduce bias into the results. Publications and citations cannot assess the content or quality of the research. It will be necessary in the future to conduct systematic reviews or meta-analyses to further evaluate the overall research quality in this field. Furthermore, there is a delay in the indexing and updating of literature, and it is necessary to update the content of bibliometric research in real time in the future [22].

## Conclusions

This study analyzed the research trends in the field of appearance anxiety through bibliometrics. The expansion of research scale in this field coexists with the dilution of academic influence. The USA is the dominant country in this field. The USA and the United Kingdom lead global collaboration. China and Australia have close internal collaboration but limited cross-regional cooperation. International collaboration urgently needs to be strengthened. The research hotspots focus on studies related to appearance anxiety and related psychological and social aspects. They are concentrated on issues related to body image and mental health among the adolescent population. The research frontiers not only delve deeper into the individual but also examine the long-term impact of appearance anxiety on an individual's social functions, interpersonal relationships, and overall quality of life.
